# Criteria for Return-to-Play (RTP) after Rotator Cuff Surgery: A Systematic Review of Literature

**DOI:** 10.3390/jcm11082244

**Published:** 2022-04-17

**Authors:** Marco Bravi, Chiara Fossati, Arrigo Giombini, Andrea Macaluso, José Kawazoe Lazzoli, Fabio Santacaterina, Federica Bressi, Ferruccio Vorini, Stefano Campi, Rocco Papalia, Fabio Pigozzi

**Affiliations:** 1 Department of Physical and Rehabilitation Medicine, Università Campus Bio-Medico, 00128 Rome, Italy; m.bravi@policlinicocampus.it (M.B.); f.santacaterina@policlinicocampus.it (F.S.); f.bressi@policlinicocampus.it (F.B.); 2 Department of Movement, Human and Health Sciences, University of Rome “Foro Italico”, 00135 Rome, Italy; arrigo.giombini@uniroma4.it (A.G.); andrea.macaluso@uniroma4.it (A.M.); fabio.pigozzi@uniroma4.it (F.P.); 3 Biomedical Institute, Medical School, Fluminense Federal University, Niterói 24220-008, Brazil; jklazzoli@uol.com.br; 4 Department of Orthopaedic and Trauma Surgery, Università Campus Bio-Medico, 00128 Rome, Italy; f.vorini@unicampus.it (F.V.); s.campi@policlinicocampus.it (S.C.); r.papalia@policlinicocampus.it (R.P.)

**Keywords:** return-to-play, rotator cuff repair, shoulder injuries, shoulder surgery, shoulder assessment

## Abstract

This systematic review of the literature aimed to highlight which criteria are described in the literature to define when a patient, after rotator cuff repair (RCR), is ready for return-to-play (RTP), which includes return to unrestricted activities, return to work, leisure, and sport activities. An online systematic search on the US National Library of Medicine (PubMed/MEDLINE), SCOPUS, Web of Science (WOS), and the Cochrane Database of Systematic Reviews, was performed with no data limit until December 2021. A total of 24 studies that reported at least one criterion after RCR were included. Nine criteria were identified and among these, the most reported criterion was the time from surgery, which was used by 78% of the studies; time from surgery was used as the only criterion by 54% of the studies, and in combination with other criteria, in 24% of the studies. Strength and ROM were the most reported criteria after time (25%). These results are in line with a previous systematic review that aimed to identify RTP criteria after surgical shoulder stabilization and with a recent scoping review that investigated RTP criteria among athletes after RCR and anterior shoulder stabilization. Compared to this latest scoping review, our study adds the methodological strength of being conducted according to the Prisma guidelines; furthermore, our study included both athletes and non-athletes to provide a comprehensive view of the criteria used after RCR; moreover, ten additional recent manuscripts were examined with respect to the scoping review.

## 1. Introduction

Rotator cuff injuries are common shoulder injuries that often cause pain and subsequent dysfunction and require surgical repair. The supraspinatus tendon is the most frequently involved, but isolated lesions of the supraspinatus tendon only occur in 40% of cases [1]. Rotator cuff repair (RCR) is currently considered as definitive treatment for rotator cuff tears and no differences have been found between arthroscopic repair and mini-open technique, which are the two main surgical techniques [2]. In the general population, the estimated prevalence of rotator cuff tears varies between 9.7% and 62% in patients aged 20 and 80, respectively [3]. Among athletes, rotator cuff injuries are quite common: the study by Kaplan et al. [4] reported that 12% of competitive collegiate American football players had a history of rotator cuff injuries. Furthermore, athletes who play overhead sports are at greater risk of injury than other athletes due to the repetitive stresses imposed on the shoulder, while athletes who play contact sports have an additional increased risk of acute traumatic injuries [5]. These injuries can be highly impacting for professional athletes and can compromise career opportunities [6,7]; therefore, it is crucial not to delay the return-to-play (RTP) in safe conditions.

The decision-making process related to RTP after RCR is complex and requires a multidisciplinary approach involving the injured athlete, physicians, physiotherapists, and the athletic training staff. However, there are currently no clear criteria to precisely define when and whether an athlete is safely ready to RTP after rotator cuff repair. One of the reasons could be the lack of reliable and valid quantitative tests and indices that can guide the choices of clinicians regarding progression through the different phases of the rehabilitation process [8,9].

A systematic review by Ciccotti et al. [10] identified seven criteria that have been used to determine whether patients, after another shoulder condition, the anterior stabilization surgery, are ready or not to RTP, and 75% of studies used the time from surgery as the only criterion. A recent scoping review by Griffith et al. [11] confirmed the results of Ciccotti et al. [10] with regard to anterior stabilization surgery, and showed the same trend in RCR studies.

The literature lacks a systematic review regarding RTP criteria used after RCR. Therefore, the aim of this systematic review of the literature is to analyze and describe what are the currently reported criteria for RTP after RCR.

## 2. Materials and Methods

### 2.1. Systematic Literature Search

An online systematic search on the US National Library of Medicine (PubMed/MEDLINE), SCOPUS, Web of Science (WOS), and the Cochrane Database of Systematic Reviews, was performed with no data limit until December 2021, according to the Preferred Reporting Items for Systematic Reviews and Meta-analysis (PRISMA) guidelines [12]. The Population Intervention Comparison and Outcome (PICO) model was adopted to conduct an evidence-based practice literature search, [13] (Table 1). The review protocol has been registered on PROSPERO (registration number: CRD42022306254).

The search strategy (Table 2) used a combination of medical subject heading (MeSH) terms and free-text terms adjusted according to each database characteristics; in addition, we performed a manual and a reference lists search.

### 2.2. Eligibility Criteria and Data Extraction

The articles that were included in this systematic review had to meet the following inclusion criteria: (1) English language full-text articles; (2) Level I to IV studies of patients undergoing surgical repair of rotator cuff tear; (3) population of patients aged ≥13 years); (4) describe criteria to RTP.

According to Ciccotti et al. [10] we defined RTP also as the return to full and/or unrestricted activity including sports, work, etc. We excluded (1) studies lacking explicit return to play criteria; (2) review articles, biomechanical studies, technical notes; (3) studies in which surgical procedures were not described.

Firstly, duplicated references were checked and excluded through the Rayyan web app [14] for systematic reviews. Eligible articles were identified independently by two reviewers (MB, CF) by screening title and abstracts, then the inclusion of all articles was discussed by the two reviewers. Subsequently, both reviewers screened the full text of the selected articles to verify if they met inclusion criteria.

Data of eligible studies were extracted, including the name of the first author, year of publication, study inclusion criteria, participants’ description; surgery technique, mean time to RTP in months, RTP criteria. Discrepancies were discussed with a third reviewer (SC). Incomplete data were treated as follows: firstly, we tried to contact the corresponding author; in case of non-response, we verified the presence of the data of interest published in other systematic reviews.

### 2.3. Quality Assessment

The methodological quality of included studies were assessed according to Ma et al. [15]. The MINORS (Methodological Index for Nonrandomized Studies) checklist [16], a specific tool developed to assess the quality of non-randomized surgical studies, was used to assess non-randomized studies. Two independent reviewers (FS, FV) assessed included studies and if discrepancies were not resolved by discussion, a third reviewer (FB) was consulted.

## 3. Results

**Figure 1:**
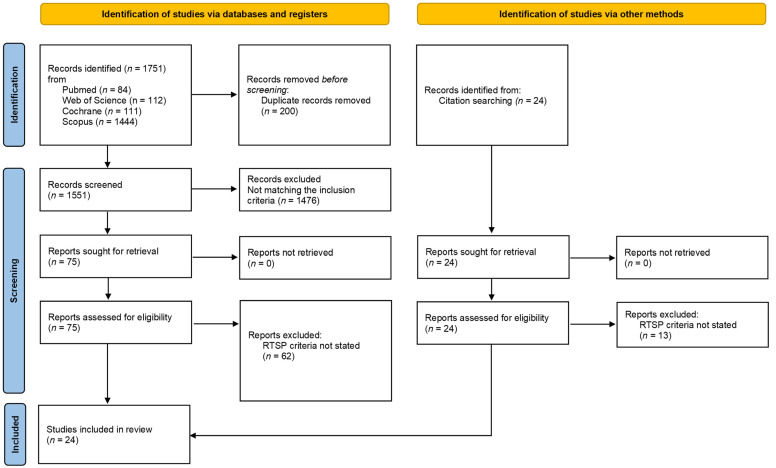
PRISMA Flowchart. From [41].

A total of 1.751 records were found, including 84 from PubMed, 1.444 from SCOPUS, 111 from the Cochrane Library, and 112 from WOS. Additionally, 24 records were found through citation searching, of which 13 records were excluded because they did not report RTP criteria. A total of 24 articles [17,18,19,20,21,22,23,24,25,26,27,28,29,30,31,32,33,34,35,36,37,38,39,40] were included in systematic review (Figure 1). The quality of the included studies is reported in Table S1; all the studies were non-comparative studies, for this reason only the items from 1 to 8 of the MINORS check list were rated according to Slim et al. [16]. Details of the included studies are summarized in Table S2.

### 3.1. Patients’ Characteristics

This systematic review included in total 847 participants (301 female and 544 male). The mean age of the participants was reported in all studies except in the study by Mazoue et al. [24] in which, however, the age of each single participant was reported and it was therefore possible to calculate mean age and standard deviation. Therefore, the mean age of the participants was of 42.8 ± 14.62 years.

Regarding the type of sport practiced, it was not possible to draw a satisfactory summary as only 11 studies [17,18,24,28,30,32,33,35,36,38,39] out of 24 accurately reported the type of sport that was practiced. Azzam et al. [29] described all sports practiced by participants; however, the number of sports exceeded the total number of patients, since some participants practiced two or three sports at the same time. Similarly, Liem et al. [23] reported that two patients practiced two sports. Among these studies, baseball is the sport most practiced by patients undergoing RCR with 88 participants (22%), followed by tennis (15%) and swimming (13%). 

### 3.2. Surgical Procedures

In most of the studies [17,18,20,22,23,26,27,28,29,30,31,32,35,37,38,39,40] the arthroscopic technique for RCR was adopted (17/24; 70.8%). In one study [21] both techniques were used depending on the lesion size: arthroscopic rotator cuff repair was performed on most patients and open rotator cuff repair was chosen when tear size was >3 cm. In one study [25] the removal of calcifications was associated with the arthroscopic technique. In four studies [19,33,34,36], the open repair technique was used; among these, in the study by Bartl et al. [34], the open technique was used for the repair of the subscapularis muscle. Mazoue et al. [24] used a mini-open repair technique.

### 3.3. Return to Play Rates, Time and Criteria

All but one study (23/24; 95.8%) reported information on RTP rates for a total of 712 patients. The average RTP rate was 88.4 ± 10.6% (range, 100% to 58.3%); 19 out of 24 studies [17,18,20,22,23,25,26,27,28,29,30,31,32,33,36,37,38,39,40] reported a RTP rate >80%, 3 studies [19,34,35] reported an RTP rate >60% and only 1 study [24] reported a rate <60%.

The time to RTP was indicated by 13 studies [18,20,21,23,24,25,27,28,32,33,38,39,40] (13/24; 54.1%) for a total of 509 patients. The mean RTP time was 7.78 ± 3.20 months (range, 14 to 4.5 months).

Regarding the surgical procedures and the RTP, in 10 studies [18,20,23,25,27,28,32,38,39,40] arthroscopic repair was performed and RTP time was on average 6.8 ± 1.7 months; in one study [21], both techniques were used depending on lesion size (see Section 3.2), and an average RTP time of 14 months was reported; in another study [24], the mini-open technique was used, and an average RTP time of 4.5 months was reported; the study by Tibone et al. [33], in which the open technique was used, reported a RTP time ranging from 12 to 18 months for baseball pitchers.The year of publication did not show any correlation with the time of RTP. 

All 24 included studies stated at least one RTP criterion. A total of nine different criteria were reported including: time, surgeon agreement, patients’ desire for RTP, sport-specific training program, range of motion, muscle strength, pain, functional recovery, and proprioception. Fourteen studies [17,19,21,22,23,30,31,33,34,36,37,38,39,40] reported only one criterion (time); four studies [20,28,29,35] reported a combination of two criteria; four studies [18,25,26,27] used a combination of three RTP criteria; two studies [24,32] reported a combination of four criteria. All the combinations of RTP criteria are reported in Table 3.

#### 3.3.1. Time

Time from surgery alone or in combination with other criteria was the most used RTP criterion. Six studies [18,20,32,35,37,40] reported that at least 3 months is the minimum time required to RTP after surgery. Two studies [17,19] reported at least 4 months and two studies [23,29] at least 5 months. Most of the studies [21,28,30,31,33,34,36,38,39] (10/20 reported time as an RTP criterion) indicated at least 6 months: among these, in two studies [33,36], RTP increased to at least 12 months for specific sport activities (serving in tennis and pitching in baseball).

#### 3.3.2. Strength

Shoulder muscle strength was indicated as a criterion for RTP in six studies [20,24,25,26,27,32]. The definition of strength level is described in a variable way: shoulder strength restoration [20], satisfactory muscle strength [24], shoulder strength near to 100% [25], shoulder strength near the same as pre-injury [26,27], and satisfactory isokinetic strength (not clearly specified) [32].

#### 3.3.3. Pain

A total of four studies [24,25,26,27] used pain as a criterion for RTP (always in combination with other criteria). Pain was defined as “non-painful ROM” [24] and “pain free” [25,26,27].

#### 3.3.4. Range of Motion

We found that six studies [24,25,26,27,32,35] reported ROM as a RTP criterion, which was used always in combination with other criteria. The ROM was defined as follows: full non-painful ROM [24], full shoulder ROM [25,26,27], satisfactory ROM (not clearly specified) [32], and Bhatia et al. [35] reported kinematic progress that we interpreted as ROM restoration.

#### 3.3.5. Specific Training Programs

Two studies [24,29] reported completion of the sport-specific training program as an RTP criterion in combination with other criteria. Azzam et al. [29] reported that patients needed to complete a sport-specific rehabilitation progression or interval training program, depending on the sport involved, to be released to full unrestricted activity. Mazoue et al. [24] reported specific criteria for pitchers (completion of two-phase interval throwing program flat-ground program and throwing from the mound), and for position players (completion of flat ground throwing program).

#### 3.3.6. Other Criteria

Tambe et al. [32] reported satisfactory proprioception (not better specified) as another criterion used to determine when the patient is ready to RTP. Antoni et al. [18] reported willingness to return and surgeon agreement as additional RTP criteria. Finally, Shimada et al. [28] reported a not better specified “functional recovery” to state readiness for RTP.

#### 3.3.7. Return to Preinjury Level and Retear

We analyzed the percentage of athletes who have returned to equal or higher levels of performance than preinjury in relation to RTP criteria. Among the 15 studies [17,18,19,21,22,23,30,31,33,34,36,37,38,39,40] that used only “time” as RTP criterion, 3 authors did not report the new level of performance, the other 12 studies reported that on average 76% (range, 42–100%) of the athletes returned to equal or higher pre-injury level. Regarding the risk of retear, four studies [36,38,39,40] reported no retear cases, Bartl et al. [34] reported three small retears, Hawkins et al. [19] reported two cases of retear, and Liem et al. [23] five cases of retear, while six studies [17,18,21,30,33,37] did not report data about retear. As for the studies that used ROM, strength, and pain as RTP, we found that the three studies by Ranalletta et al. [25] and Rossi et al. [26,27] reported that an average 83.7% (range, 80–91.3%) of athletes returned to an equal or higher pre-injury level with no cases of retear. The other studies combining multiple criteria for RTP reported an average return to performance of 62% (range, 21–93%), and only two studies (Azzam et al. [29] and Shimada et al. [28]) reported one case and three cases of retear, respectively.

## 4. Discussion

The main finding of this systematic review, which aimed at identifying criteria for RTP following rotator cuff surgery, was that nine criteria were identified and “time from surgery” was the only criterion in 54% of the studies, and was the most reported criterion in 78% of the studies, followed by “strength and ROM”, which were reported in 25% of the studies. In most cases, no clear rationale is given for using certain criteria over others. Therefore, there is a need to study and validate a set of RTP objective criteria with the aim of providing evidence-based indications that can be useful in clinical practice to define patient readiness. Furthermore, when dealing with athletes, criteria about the athlete’s ability to perform sport-specific gestures should also be considered. The major strength of this systematic review in comparison with previous studies is the methodological rigour in applying Prisma guidelines and the inclusion of both athletes and non-athletes.

Regarding RTP criteria, some considerations are required. The time from surgery is the most widely used criterion by the included studies, with an average of 6–7 months for RTP. We believe that time from surgery must certainly be considered during rehabilitation after RCR to guide the progression of rehabilitation treatments and exercises, but it cannot be the only criterion used to define readiness for RTP. The studies did not clarify the reason behind the choice of this interval of time for RTP, which can likely be attributed to preserving the integrity of the repaired tendon. However, Sonnabend et al. [42], in a study on a primate model, showed that after 15 weeks the bone-tendon junction was almost mature indicating that up to 15 weeks rehabilitation programs should protect surgical repair. Therefore, after an adequate safety time has been exceeded, other criteria such as strength, ROM, and function, should guide the choice of clinicians to define patient’s readiness for RTP. Kibler et al. [43] pointed out that it would be appropriate to establish specific criteria especially for the return to sport, which require the recovery of functional capacity and should be objectively demonstrated through the measurement of ROM, strength, and through physical performance tests, rather than being evaluated solely based on time from surgery or imaging. Results from this systematic review evidenced that the included studies, published from 1986 to 2021, reported a time from surgery that remained substantially unchanged, especially if we consider the studies of the last 15 years. Therefore, despite the progress in surgery and rehabilitation fields, the “time” criterion (usually reported to be at least of 6 months) appears to be handed down over the years in a quite empirical way, and is barely supported by human and animal models.

The strength criterion, and more precisely the level of strength, has never been adequately defined; in fact, most studies do not mention how strength is assessed (i.e., maximum voluntary isometric strength, manual muscle strength testing, or isokinetic strength assessment). As a matter of fact, only the study by Tambe et al. [32] reported the use of isokinetic evaluation, without reporting the levels of strength. A recent consensus statement [44] stated that all sports with demands on the shoulder have a shoulder strength requirement and the external rotation/internal rotation (ER/IR) strength ratios should be used as a criterion for RTP. According to Thigpen et al. [45] it would be safe to assess muscle performance 4 months after surgery since several studies [5,45,46,47], in which strength was assessed with a handheld dynamometer after 4 months, reported no injuries. However, further studies should specify threshold absolute values of strength, as the use of the ER/IR ratio, as suggested by Schwank et al. [44, is not indicative of readiness for RTP, since an athlete who is weak could have a normal ER/IR ratio. These thresholds should be related to preinjury measurements, when available, or to healthy controls or contralateral limb. Furthermore, as reported by Pigozzi et al. [48], it would be useful to measure strength also during dynamic movements, since instrumented stacked plate resistance machines, which have a high test–retest reliability, allow muscle power to be determined, which is more strongly associated with functional abilities and sports performance than strength per se.

In accordance to the scoping review by Griffith et al. [11], our systematic review found a total absence of patient-reported outcome measures (PROMs) among RTP criteria, without a clear explanation of why PROMs were not used as RTP criteria. However, as highlighted in the recent 2022 Bern Consensus [44], it should be recommended to use shoulder-specific PROMs (i.e., Shoulder Pain and Disability Index, American Shoulder and Elbow Surgeons standardized shoulder assessment form, etc., see Appendix C of 2022 Bern Consensus Statement on Shoulder Injury Prevention, Rehabilitation, and Return to Sport for Athletes at All Participation Level), and to define cut-off levels to be used in conjunction with other criteria to determine when a patient is ready for RTP.

All the studies that have reported ROM as a criterion for RTP frequently required a full ROM recovery before allowing RTP; however, the use of this criterion can limit a timely return to sport, since although the overhead athletes need full ROM before return to sport, there is no need of full ROM restoration for collision athletes [44]. Therefore, it would be desirable that future studies be more specific in the use of this criterion and clarify the reason for the choice; it may be more useful to define this criterion as sport/activity-specific functional ROM recovery rather than generic full ROM recovery.

Pain is another domain that should certainly be taken into consideration to allow the RTP, but, also for this criterion, it is necessary to make essential distinctions. In fact, the total absence of pain is not required for the general population to return to unrestricted activities, while on the contrary all the athletes who have to return to sports activities and to preinjury performances must be pain-free [44].

The study by Tambe et al. is the only one that reported an improvement of shoulder proprioception as a criterion to RTP, without specifying the assessment modality. Active shoulder proprioception [49] should be considered as a RTP criterion; as shown by Gumina et al. [50], a rotator cuff tear causes an alteration of the joint position sense, which consequently results in a reduction in the neuromuscular control, with the latter being essential for an athlete’s return to competition [51]. Finally, the results of this systematic review show the total absence of psychological readiness, which is a not negligible factor, especially when deciding whether the athlete is ready for the RTP; fear of re-injury and motivation may influence treatment and readiness to RTP after an injury [52]. Therefore, some scales such as the Tampa Scale of Kinesiophobia [53] or the Injury–Psychological Readiness to Return to Sport scale (I-PRRS) [54] should be used in association with other criteria.

Finally, there is no clear correlation between the use of defined RTP criteria and return to performance after RCR. As a matter of fact, the data from the studies grouped by RTP criteria show values that are close to the total average of 73% of athletes returning to equal or higher preinjury levels. Therefore, the use of different RTP criteria does not seem to influence the ability to return to preinjury levels; indeed, as also reported by Altintas et al. [55], we believe that the ability to return to performance is multifactorial: preinjury sports participation (recreational or competitive), type of sport (overhead, collision), age, etc., can all influence the return to preinjury levels.

The following limitations of this systematic review need to be highlighted. Firstly, we are not sure that the description of the criteria has been carried out in an exhaustive way; as pointed out by Ciccotti et al. [10] and by Griffith et al. [11], it is likely that some studies have omitted more accurate criteria descriptions due to publication-related limits. Our review included both athletes and non-athletes, which allows for the inclusion of a greater number of studies, but, on the other hand, makes the sample more heterogeneous; we only included English written studies, and it is therefore likely that some studies written in a different language were not considered.

## 5. Conclusions

Our review identified a total of nine criteria that have been used in the literature to determine the patient’s readiness to RTP after RCR. Consistently with the examined papers, we have found that the time from surgery is the most widely used standard. The use of additional criteria is desirable in future studies, as we believe that it is not enough to decide when a patient is ready for the RTP solely on time and strength criteria. Future studies should strive to use criteria about shoulder function and proprioception, since emerging technologies now offer clinicians low-cost precise and reliable measurement tools (i.e., wearable magneto-inertial sensors, dynamometers, and load cells), which allow a complete assessment of the shoulder function to be easily performed [56]. Finally, when dealing with an athlete, the athlete’s perception and psychological readiness should be included among RTP criteria, as psychologic factors are associated with longer duration of symptoms and higher levels of disability [57] that can negatively impact shoulder function during sport-specific gestures.This will hopefully lead to the determination of a set of activity/sport-specific conditions that can be used to correctly establish readiness for RTP, ensuring the safety of patients and avoiding reinjuries.

The following bullets points summarize the results of this systematic review:

- “Time from surgery” is the most used criterion to define readiness for RTP.
- Strength recovery is rarely used and poorly detailed.
- Preinjury performance levels and injury rates do not appear to be related to the use of specific RTP criteria
- Despite their importance, no clinical studies have used specific PROMS and psychological readiness assessment as RTP criteria.

## Figures and Tables

**Table 1:** PICO model.

Population	Intervention	Comparison	Outcome
Patients with rotator cuff tear	Rotator cuff repair Rotator cuff surgery	-	Return to play criteria, return to unrestricted activity criteria

**Table 2:** Search strategy.

Database	Search Terms
PubMed	(“rotator cuff repair” [All Fields] OR “rotator cuff surger *” [All Fields] OR “rotator cuff tear” [All Fields] OR “Rotator Cuff Injuries” [MeSH Terms] OR “Rotator Cuff” [MeSH Terms]) AND (“return to sport” [All Fields] OR “return to play” [All Fields] OR “unrestricted activity” [All Fields] OR “full activity” [All Fields])
SCOPUS	ALL (“rotator cuff repair” OR “rotator cuff surger *” OR “rotator cuff tear” OR “Rotator Cuff”) AND (“return to sport” OR “return to play” OR “unrestricted activity” OR “full activity”)
Cochrane	(rotator cuff repair OR rotator cuff surger OR rotator cuff tear) AND (return to sport OR return to play OR unrestricted activity OR full activity)
WOS	(ALL = (“rotator cuff repair” OR “rotator cuff surger *” OR “rotator cuff tear” OR “Rotator Cuff”)) AND ALL = (“return to sport” OR “return to play” OR “unrestricted activity” OR “full activity”)

* asterisk is added to perform truncation search.

**Table 3:** Combinations of RTP criteria among included studies.

Combination of RTP Criteria	Number of Studies (%)
Time	14 (58%)
Time, sport specific training program	1 (4%)
Time, ROM	1 (4%)
Time, strength	1 (4%)
Time, functional recovery	1 (4%)
Time, surgeon agreement, patients’ desire	1 (4%)
ROM, strength, pain	3 (13%)
ROM, strength, pain, sport specific training program	1 (4%)
Time, ROM, strength, proprioception	1 (4%)

## Supplementary Materials

The following supporting information can be downloaded at: https://www.mdpi.com/article/10.3390/jcm11082244/s1, Table S1: Quality assessment; Table S2: Details of included studies.
