# Supplementary material for: Criteria for Return-to-Play (RTP) after Rotator Cuff Surgery: A Systematic Review of Literature
Source: J Clin Med. 2022 Apr 17;11(8):2244. doi: 10.3390/jcm11082244 (PMC9024603; doi:10.3390/jcm11082244)
Supplement: Supplementary file 1 [file jcm-11-02244-s001.zip › jcm-1661617-supplementary.pdf]

Journal of
Clinical Medicine

Table S1. Quality assessment.

	Clearly defined aim	Consecutive patients	Prospective data collection	Adequate endpoint	Blind assessment	Adequate length of follow-up	Loss to follow-up rate < 5%	Prospective calculation of the study size	Total score
Andrews et al. (1985)	2	2	0	1	0	2	0	0	7/16
Antoni et al. (2016)	2	2	0	1	0	2	2	0	9/16
Azzam et al. (2018)	2	2	0	1	0	2	2	0	9/16
Bartl et al. (2010)	2	2	0	1	2	2	2	0	11/16
Bhatia et al. (2015)	1	2	1	1	0	2	1	0	8/16
Bigiliani et al. (1992)	2	2	0	1	0	2	2	0	9/16
Burns et al. (2008)	2	2	0	1	0	2	2	0	9/16
Carbone et al. (2020)	2	2	2	1	1	2	2	0	12/16
Carbone et al. (2021)	2	2	2	1	2	2	2	2	15/16
Davey et al. (2021)	2	2	0	1	0	2	2	0	9/16
Hawkins et al. (1999)	2	2	0	1	0	2	2	0	9/16
Ide et al. (2005)	2	2	2	1	1	2	2	0	12/16
Kim et al. (2019)	2	2	2	1	0	2	2	2	13/16
Krishnan et al. (2008)	1	2	0	1	0	2	1	1	8/16
Liem et al. (2008)	2	2	2	1	0	2	2	0	11/16
Mazoue et al. (2006)	2	2	1	1	0	2	2	0	10/16
Ranalletta et al. (2016)	2	2	1	1	0	2	2	0	10/16
Rossi et al. (2019a)	2	2	1	1	2	2	2	0	12/16
Rossi et al. (2019b)	2	2	1	1	2	2	2	0	12/16
Shimada et al. (2020)	2	2	2	1	0	2	1	0	10/16
Simon et al. (2017)	2	1	0	1	0	1	2	0	7/16
Spencer (2010)	2	1	0	1	0	2	2	0	8/16
Tambe et al. (2009)	2	1	0	1	0	2	2	0	8/16
Tibone et al. (1986)	2	2	1	1	0	2	2	0	10/16

Table S2. Details of included studies.

Author (year)	Design and Aim	Participants	Type of sport	Surgery procedure	Patients at follow up n (% of included)	Mean RTP Time and rates	RTP reported Criteria
Andrews et al. (1985)	Retrospective<br><br>To reports preliminary experience with arthroscopy as an aid in the diagnosis and treatment of patients with partial tears of the rotator cuff.	36 patients. Female n = 3; Male n = 33. Mean age = 22 (range, 16-29) years.	Baseball (n = 27); football (n = 7); tennis (n = 2).<br><br>All patients were involved in competitive athletic activities.	Arthroscopic repair	34/36 (94.4%)<br><br>Evaluation mean FU = 13.1 months.	Time not stated<br>RTP rate = 85%	- Time (4 to 6 months)
Antoni et al. (2016)	Retrospective single-center study<br><br>to assess return to sports in recreational athletes after arthroscopic repair of rotator cuff tear (RCT)	76 patients. Female n = 37.<br>Male n = 39.<br>Mean age = 57 ± 7.3 years	Tennis, swimming, physical exercise, and golf.	Arthroscopic RCR for unilateral full-thickness rotator cuff tear	67/76 (88.1%)<br><br>Evaluation mean FU = 45 ± 14 months.	Time = 6 ± 4.9 months<br>RTP rate = 88.2%	- Time (3 months)<br>- Surgeon agreement<br>- Desire of the patient
Azzam et al. (2018)	Case-series<br><br>to investigate the clinical information, demographics, and results of surgical treatment for a consecutive series of adolescent athletes who underwent rotator cuff repair with a minimum 2-year follow-up.	32 adolescent patients. Female n = 4; Male n = 28.<br>Mean age = 16.1 ± 1.3 years	Football, baseball, basketball, softball and wrestling.	Arthroscopic repair with a single- or double-row configuration.<br>Open approach with suture anchors and a double-row construct for subscapularis injuries.	27/32 (84.3%)<br><br>Evaluation mean FU = 6.2 ± 2.6 years.	Time not stated<br>RTP rate = 93%	- Time (5 to 6 months for sport-specific exercise)<br>- Achievement of sport-specific or interval training program
Bartl et al. (2010)	Case-series<br><br>To report the clinical outcome, value of clinical tests, predictive outcome factors, and return to sports in the management of isolated traumatic subscapularis tendon tears	30 patients. Female n = 4; Male n = 26.<br>Mean age = 43.1 (range, 15-64) years.	28% were involved in contact sports (soccer, handball, ice hockey); 32% in overhead sports (tennis, volleyball, gymnastics, climbing) and 40% in other sports (water skiing, alpine skiing, cycling, equestrian, and others)	Open subscapularis repair	30/33 (91%)<br><br>Evaluation mean FU = 46 (range, 25-72) months.	Time not stated<br>RTP rate = 75%	- Time (6 months)

Bhatia et al. (2015)	Case-series<br><br>To evaluate clinical outcomes after arthroscopic repair of full-thickness rotator cuff tears in recreational athletes aged 70 years or older	44 patients (49 shoulders). Female n = 11; Male n = 33. Mean age = 73 ± 3.2 years.<br><br>The most frequent sports practiced before surgery were	Alpine skiing, golf, other sports such yoga, fitness, swimming.	Arthroscopic repair with a single- or double-row configuration.	43/49 shoulder (87.7%)<br><br>Evaluation mean FU = 3.6 (range 2.0-7.9) years.	Time not stated<br>RTP rate = 77%	- Time (3 to 4 months)<br>- Kinematic progress
Bigiliani et al. (1992)	Observational retrospective<br><br>to report the results of anterior acromioplasty and rotator cuff repair in a group of tennis players in whom one of the primary goals was to return to playing tennis.	23 patients. Female n = 6. Male n = 17. Mean age = 58 (range, 39-71) years	Tennis (n = 23)	Open repair	23/23 (100%)<br><br>Evaluation mean FU = 39 (range, 18-109) months	Time not stated<br>RTP rate = 96%	- Time (6 to 12 months)
Burns et al. (2008)	Retrospective<br><br>to report the results of arthroscopic rotator cuff repairs in a younger population and to analyze the results by tear type, associated pathology, and patient demographics	37 patients (41 shoulders). Female n = 13. Male n = 24. Mean age = 43 (range, 30-49) years.	12 patients participated in overhead athletics (i.e., tennis, ball sports, swimming). Remaining patients were not involved in sports.	Arthroscopic repair.	37/37 (100%)<br><br>Evaluation mean FU = 5.8 (range, 3.1-13.4) years.	Time not stated<br>RTP rate = 80%	- Time (3-4 months)
Carbone et al. (2020)	Case-series<br><br>to evaluate the results of arthroscopic rotator cuff repair in a consecutive series of athletes and their consequent return to CrossFit activities.	22 patients (23 shoulders); Female n = 2; Male n = 20. Mean age = 40.5 ± 8.8 years.	Cross-Fit (n = 22)	Arthroscopic repair. Mini-open tenodesis in case of complete rupture of the biceps with a positive Popeye sign.	22/22 (100%)<br><br>Evaluation mean FU = 24 months.	Time = 8.7 ± 3.4 months<br>RTP rate = 100%	- Time (6 months)

Carbone et al. (2021)	Case-series<br><br>to report clinical and MRI results of the arthroscopic repair of isolated supraspinatus tear associated with SLAP lesion in competitive CrossFit athletes, to assess if they have a different timing for return to sport.	19 patients; Female n = 7; Male n = 12. Mean age = 43 ± 8 years.	Cross-Fit (n = 19)	Arthroscopic repair.	19/19 (100%)<br><br>Evaluation mean FU = 24 months.	Time = 9.8 ± 4 months<br>RTP rate = 100%	- Time (6 months)
Davey et al. (2021)	Retrospective<br><br>To evaluate clinical outcomes and rate of RTP among athletes aged 30 years or younger who have undergone an arthroscopic rotator cuff repair after trauma.	20 patients. Female n = 4; Male n = 16. Mean age = 25.5 (range 18-29) years.	Type of sport were not stated. 75% of participants were collision athletes and 20% were overhead sport athletes.	Arthroscopic repair with a double-row configuration.	20/20 (100%)<br><br>Evaluation mean FU = 31.8 ± 14.7 months	Time = 5.8 ± 2.8<br>RTP rate = 85%	- Time (3 to 6 months)
Hawkins et al. (1999)	Retrospective<br><br>To analyze the possible contributing factors, associated clinical ramifications, and surgical results of full-thickness rotator cuff tears in patients younger than 40 years of age.	19 patients. Female n = 2; Male n = 17. Mean age = 33.4 (range 23-40) years	Not stated	Open repair.	19/19 (100%)<br><br>14 returned to work, of which 9 returned to sports activities<br><br>Evaluation mean FU = 5.7 (range 2.1 to 12) years	Time not stated<br>RTP rate = 73.6%	- Time (4 to 6 months)
Ide et al. (2005)	Case-series<br><br>To investigate the outcomes in patients treated by arthroscopic transtendon repair in patients with significant partial thickness articular-side rotator cuff tears	17 patients. Female n = 3; Male n = 14. Mean age = 42 (range 17-51) years.	6 participants were overhead-throwing athletes: badminton, tennis, baseball, volleyball.	Arthroscopic repair	5/6 athletes (83.3%)<br><br>Evaluation mean FU = 39 (range 25 to 57) months	Time = 8.4 (range 6-12) months.<br>RTP rate = 83.3%	- Time (3 to 6 months)<br>- Shoulder strength restoration.

Kim et al. (2019)	Prospective<br><br>To assess when patients can perform low-level and high-level front-of-body motion, low-level and high-level behind-the-back motion, strength-related activities, and sports/leisure activities after arthroscopic rotator cuff repair.	135 patients. Female n = 78; Male n = 57.<br>Mean age = 60 ± 8 years<br><br>General population	Not stated	Arthroscopic repair. Open rotator cuff repair when tear size is > 3 cm	135/135. (100%)<br><br>Evaluation mean FU = 2 years.	Time = 14 ± 0 months<br>RTP rate not stated.	- Time (3 months for light sports activities; 6 months for all activities that place heavy demands on the shoulders).
Krishnan et al. (2008)	Retrospective<br><br>To retrospectively report results of arthroscopic repair of full-thickness RCT in patients younger than 40 years	23 patients. Female n = 8; Male n = 15.<br>Mean age = 37 (range, 21-39) years.	Not stated.	Arthroscopic repair.	23/23 (100%)<br><br>Evaluation mean FU = 26 (range, 24-29) months	Time not stated.<br>RTP rate = 90%	- Time (6 months)
Liem et al. (2008)	Retrospective<br><br>To assess if arthroscopic rotator cuff repair of isolated supraspinatus tears allows patients to return to their preinjury levels of overhead-throwing sports.	21 patients. Female n = 7; Male n = 14.<br>Mean age = 58.9 (range, 46-68) years.	Tennis (n = 11); golf (n = 5); volleyball (n = 3); swimming (n = 2); fencing (n = 1); handball (n = 1).<br>Two patients participated in 2 sporting activities.	Arthroscopic repair	21/21 (100%)<br><br>Evaluation mean FU = 25.7 (range, 24-29) months	Time = 6.3 (range, 3-12) months.<br>RTP rate = 100%	- Time (21 weeks)
Mazoue et al. (2006)	Retrospective case-series<br><br>to determine if baseball players could return to this same high level of competition after repair of a full-thickness rotator cuff tear.	16 patients. Male n = 16.<br>Mean age = 29.4 ± 3.3	Baseball (n = 16)	Mini-open repair.	16/16 (100%)<br><br>Pitchers. Evaluation mean FU = 67 (range, 33-93) months<br><br>Position players. Evaluation mean FU = 30.5 (range, 7-54) months.	Not stated for pitchers.<br>4.5 months for position players.<br>RTP rate = 58.3%	- Full, nonpainful ROM<br>- Satisfactory muscle strength<br>Pitchers:<br>- completion of 2-phase interval throwing program (flat-ground program and throwing from the mound)<br>Position players:<br>- completion of flat ground throwing program

Ranalletta et al. (2016)	Retrospective<br><br>To analyse the time to return to sport, clinical outcomes, and complications of complete arthroscopic removal of intratendinous calcific deposits and repair of the tendon lesion without acromioplasty in athletes	24 patients. Female n = 14; Male n = 10. Mean age = 36 ± 6.1 years.	13 patients were involved in non-collision/non-overhead sports; 5 patients were involved in high impact/collision; 6 patients were involved in overhead sports.	Arthroscopic repair + calcific deposits removal.	24/24 (100%)<br><br>Evaluation mean FU = 59 (range, 24-108) months.	Time: 5.3 (range, 3-9) months. RTP rate = 95.8%	- pain free<br>- full shoulder ROM<br>- shoulder strength near to 100%.
Rossi et al. (2019a)	Retrospective case-series<br><br>To evaluate return to sport, clinical outcomes, and complications in a series of athletes with painful partial thickness rotator cuff tears treated with the arthroscopic in situ repair with a minimum 2-year follow-up	72 patients. 70 patients completed FU: female n = 33. Male n = 37. Mean age = 42.2 years (range, 21-66 years).	35 patients were involved in non-collision/non-overhead sports; 20 were involved in high impact/collision sports; 15 in overhead sports.	Arthroscopic repair	70/72 (97.2 %)<br><br>Evaluation mean FU = 54 (range, 24-113) months.	Time = 5.6 (range, 3-9) months. RTP Rate = 87%	- Pain free<br>- Full-shoulder ROM<br>- shoulder strength near the same as pre-injury
Rossi et al. (2019b)	Retrospective<br><br>To analyse clinical outcomes, return to sports, and complications in a series of patients with painful partial thickness rotator cuff tears treated with arthroscopic in situ repair with suture anchors who had a minimum of 8 years of follow-up.	62 patients. Female n = 27. Male n = 35. Mean age = 52.4 (range, 32-67) years.	21 were recreational athletes and 9 were competitive Athletes. Type of sports not stated.	Arthroscopic repair	62/62 (100%)<br><br>Evaluation mean FU = 10.4 (range, 8-12) years.	Time not stated. RTP rate = 87%	- Pain free<br>- Full-shoulder ROM<br>- shoulder strength near the same as pre-injury
Shimada et al. (2020)	Retrospective case-series<br><br>To investigate clinical outcomes and return to sport after arthroscopic rotator cuff repair in middle-aged and elderly swimmers	31 patients (32 shoulders). Female n = 26. Male n = 5. Mean age = 65 (range, 47-78) years.	Swimming (n = 31)	Arthroscopic repair	32/36 (89%)<br><br>Evaluation mean FU = 47 (range, 24-86) months.	Time = 8 (range, 3-24) months. RTP rate = 97%	- Time (6 months)<br>- Functional recovery (not clearly reported).

Simon et al. (2017)	Retrospective<br><br>To analyze the general (Constant Murley score) and sports-specific outcome after surgical repair of rotator cuff injuries in rock climbers.	12 patients. Female n = 2. Male n = 10.<br>Mean age = 55 ± 9 years.	Rock-climbing (n = 12)	Arthroscopic repair	12/12 (100%)<br><br>Evaluation mean FU = 27 ± 16 months.	Time not stated.<br>RTP rate = 100%	- Time (6 months)
Spencer (2010)	Retrospective<br><br>To compare the preoperative and postoperative Penn shoulder scores (PSS) associated with an in situ all-inside repair technique and the effects of such a repair on postoperative stiffness	20 patients. Female n = 4. Male n =16.<br>Mean age = 41 (range, 18-54) years.	There were 2 high school athletes, the rest were recreational athletes. Type of sports not stated.	Arthroscopic repair	20/20 (100%)<br><br>Evaluation mean FU = 29 (range, 16-41) months.	Time not stated.<br>RTP rate = 95%	- Time (6 months).
Tambe et al. (2009)	Retrospective<br><br>To investigate the results and functional outcomes of arthroscopic rotator cuff repairs in elite rugby players only over a two-year period.	11 patients. Male n = 11.<br>Mean age = 25.7 (range, 19- 31) years.	Rugby (n = 11)	Arthroscopic repair	11/11 (100%)<br><br>Evaluation mean FU = 18 (range, 12-31) months.	Time = 4.8 (range, 3-8) months.<br>RTP rate = 100%	- Time<br>- Satisfactory ROM (not clearly specified)<br>- Satisfactory isokinetic strength (not clearly specified)<br>- Satisfactory proprioception (not clearly specified)
Tibone et al. (1986)	Retrospective<br><br>To present the results of the surgical treatment of tears of the rotator cuff in athletes.	45 patients. Female n = 6. Male n = 39.<br>Mean age = 29 (range, 18-40) years.	Baseball (n = 29); tennis (n = 4); volleyball (n = 2); swimming (n = 2); javelin-throw (n = 2); other sports (n = 6).	Open repair	45/45 (100%)<br><br>Evaluation mean FU = 3.5 (range, 2-6) years.	Time = 12-18 months for pitchers.<br>RTP rate = 86.6%	- Time (6 months for non-pitchers; 12 months for pitchers).

FU = follow up; ROM = range of motion.
